# Comparative Genetic Association Analysis of Human Genetic Susceptibility to Pulmonary and Lymph Node Tuberculosis

**DOI:** 10.3390/genes14010207

**Published:** 2023-01-13

**Authors:** Abhimanyu, Mridula Bose, Astha Giri, Mandira Varma-Basil

**Affiliations:** 1 Department of Microbiology, Vallabhbhai Patel Chest Institute, University of Delhi, Delhi 110007, India; 2 The Global Tuberculosis Program, Baylor College of Medicine, William T Shearer Center for Immunobiology, Texas Children’s Hospital, Houston, TX 77004, USA

**Keywords:** pulmonary tuberculosis, lymph node tuberculosis, extra-pulmonary tuberculosis, single nucleotide polymorphisms, cytokine, innate immunity, genetic association, genotype, serum

## Abstract

Background: Tuberculosis (TB) manifests itself primarily in the lungs as pulmonary disease (PTB) and sometimes disseminates to other organs to cause extra-pulmonary TB, such as lymph node TB (LNTB). This study aimed to investigate the role of host genetic polymorphism in immunity related genes to find a genetic basis for such differences. Methods: Sixty-three, Single nucleotide polymorphisms (SNPs) in twenty-three, TB-immunity related genes including eleven innate immunity (*SLCA11*, *VDR*, *TLR2*, *TLR4*, *TLR8*, *IRGM*, *P2RX7*, *LTA4H*, *SP110*, *DCSIGN and NOS2A*) and twelve cytokine (TNFA, *IFNG*, *IL2*, *Il12*, *IL18*, *IL1B*, *IL10*, *IL6*, *IL4*, *rs1794068*, *IL8 and TNFB*) genes were investigated to find genetic associations in both PTB and LNTB as compared to healthy community controls. The serum cytokine levels were correlated for association with the genotypes. Results: PTB and LNTB showed differential genetic associations. The genetic variants in the cytokine genes (*IFNG*, *IL12*, *IL4*, *TNFB* and *IL1RA* and *TLR2*, *4* associated with PTB susceptibility and cytokine levels but not LNTB (*p* < 0.05). Similarly, genetic variants in *LTA4H*, *P2RX7*, *DCSIGN* and *SP110* showed susceptibility to LNTB and not PTB. Pathway analysis showed abundance of cytokine related variants for PTB and apoptosis related variants for LNTB. Conclusions: PTB and LNTB outcomes of TB infection have a genetic component and should be considered for any future functional studies or studies on susceptibility to pulmonary and extra-pulmonary TB.

## 1. Introduction

Tuberculosis (TB), a major health hazard worldwide, is characterized by different clinical manifestations including localized infection in the lungs or pulmonary TB (PTB) and various forms of extra-pulmonary (EPTB). PTB accounts for 80% of all forms of TB [1], while EPTB constitute about 15–20% of all immunocompetent TB cases and 50% in cases infected with Human Immunodeficiency Virus (HIV) [2]. The most common form of EPTB is tubercular lymphadenitis (LNTB) with 50% of the cases involving the peripheral lymph nodes [3]. The basis of the variability of disease manifestation by the same infectious organism is unclear. It is not well understood as to why some individuals have EPTB disease which can infect other sites such as lymph nodes, while most persons have localized infection in the lungs.

The propensity for such different manifestations can be attributed to environmental exposures, pathogen virulence traits and host genetics of immune response. It is not really understood which of the aforementioned factors is the most important. India being an endemic country for tuberculosis with highest number of incident TB cases in 2021 [1], the prominent role of environmental exposures would most likely not be a driving factor in this population. As for pathogen virulence traits, there is association between infectivity of *Mycobacterium tuberculosis* (*Mtb*) strain and extra-pulmonary infections [4]. Pathogen variance can differ in disease presentations and *Mtb* strains in EPTB show mutations as in the *pncA* gene [5], or insertions and deletions in the phospholipase-C gene D (*plcD*) gene [6], which is not seen strains isolated from PTB patients. To, that angle, in north India the most commonly circulating strain is Central Asian (CAS-1), Delhi and the East African Indian (EAI) strains [7], which have not been shown to be associated with either PTB or EPTB alone. Although *Mtb* strains show genetic diversity, in various manifestations of TB like PTB or meningeal TB, the association is not lineage-specific [8], pointing to the decisive nature of host-genetics in such a scenario.

Both host and pathogen genotypes can be important as *Mtb* lineage specific association with disseminated disease of tubercular meningitis was identified to be associated with the *TLR2* polymorphism. [9]. The question remains as to why individuals infected with genotypically same, or different circulating strains of *Mtb* show different immune response. As far as the host genetics is concerned, ethnic specificity of the immune response in TB has been demonstrated [10].

So, to address the question of existing variability in the important immune response genes and their effect on governing this differential manifestation of TB, we designed a comparative association study between PTB and LNTB. Host genetic association studies on different manifestations of tuberculosis are few (11–21) and comparative studies limited. EPTB studies have focused on tubercular meningitis (TBM) [11,12], pleural TB, mixed cases with any form of EPTB together [13] but not LNTB. Genetic variants in EPTB have been studied only in limited number of genes including *TLR2* [11], *IL10*, *IFNG* [13], *VDR* [14,15], *P2RX7* [16,17], *SP110* [18,19], *IL1B, IL1RA* [15,20] and a pilot scale genome-wide association study [21], while PTB has been extensively studied across multiple genes [22].

Therefore, to maximize the number of genes compared between PTB and LNTB, in this study, we have considered multiple genes in a candidate gene approach. A total of twenty-three genes which have been shown to be important in tuberculosis immunopathogenesis [22] were genotyped for 63 human genetic variants encompassing both innate and adaptive branch of tuberculosis in order to investigate both the arms of immunity.

We found that polymorphisms in the cytokine genes were associated with susceptibility to PTB. These cytokine genetic variants showed no or weaker association with LNTB. The cytokine genetic variants also showed high degree of gene-gene interaction among them accentuating their importance in governing susceptibility to PTB in north Indians. Interestingly, the variants in *SP110* [23] and *P2RX7* [24], which control macrophage apoptosis during tuberculosis infection, were associated with the risk of developing LNTB.

Overall, the study described here demonstrates that differences in host genetics is associated with different manifestations of tuberculosis. The study contributes to the emerging knowledge of key players in host-pathogen interaction in tuberculosis. We show that host genotype is a key determinant of the outcome of host-pathogen interaction and thus manifestation of tuberculosis as pulmonary and LNTB form.

## 2. Materials and Methods

### 2.1. Study Population

Venous blood Samples were collected from TB hospitals in and around New Delhi between 2009–2011. TB cases were 15 years or older culture confirmed or clinically diagnosed PTB cases with sputum smear microscopy for acid-fast bacilli (AFB), culture and chest X-ray data. Individuals included were not on any anti-tubercular therapy. For LNTB, patients were carefully selected to only have peripheral lymph node tuberculosis. Fine needle aspiration cytology (FNAC) was used to make histological confirmation of granulomatous structure and the FNAC was stained for AFB. Either histologically confirmed or AFB positive patients were considered for the LNTB group of the study. Patients with mixed EPTB and PTB infection were excluded from the study. All the enrolled patients were HIV negative. HIV negative community controls were enrolled from in and around New Delhi. The controls were confirmed to have never been diagnosed with TB and had no family history of TB. For the discovery cohort for cytokine genes, we enrolled Pulmonary TB (PTB), *n* = 110; Lymph Node TB, *n* = 35; Healthy controls, *n* = 78. For the validation cohort for 9 SNPs from six cytokine genes we had a sample size of PTB, *n* = 160, LNTB, *n* = 50: HC, *n* = 265. This was obtained by obtaining the genotypes for additional, ethnicity matched controls (*n* = 135), were added for the validation phase analysis and were obtained from the Indian genome variation consortium database [25]. For the Innate Panel we had PTB, *n* = 125; LNTB, *n* = 50 and HC *n* = 125.

All patients and volunteers were informed about the study and an informed written consent was obtained from all the study participants. This study (ID: 60(0081)/07/EMR-II), was approved by the Institutional ethics committee of Vallabhbhai Patel Chest Institute, University of Delhi.

### 2.2. SNP Selection

For the discovery panel, thirty-nine SNPs from twelve cytokine genes were selected. The SNPs mostly were in the intronic, exonic and 3′UTR regions. To avoid selection of non-polymorphic loci, as there was no data available on the Indian population at the time of the study initiation; we relied on the HapMap database (www.hapmap.org accessed on 3 November 2022). SNPs were selected based on the following criteria: 1. Reported frequency >10% in at least 3 world population in HapMap database; 2. Reported frequency >20% in at least 2 world populations in HapMap database; 3. Average heterozygosity, which is a measure of genetic diversity at population scale was considered from dbSNP (http://www.ncbi.nlm.nih.gov/projects/SNP accessed on 3 November 2022). It indicates the average proportion of individuals which are heterozygous in dbSNP from all the SNP data submitted to it, and this reduces selection on non-polymorphic loci. We have successfully implemented this strategy previously identifying novel genetic associations for TB [26]. New associations identified from the discovery panel in the study, were intersected with previously identified strong association with PTB [26] and used for the validation panel.

### 2.3. SNP Genotyping

Genomic DNA was extracted using QIAamp DNA kit (Qiagen, Hilden, Germany). The concentrations of the DNA samples were determined by Nanodrop using Nanoquant^TM^ plate of Infinite^®^ Pro 200 system (Tecan, Männedorf, Switzerland), checked for purity on an 1% agarose gel and stored at −20 °C until further analyses. All the cytokine SNPs were genotyped using the matrix-assisted laser desorption/ionization time-of-flight (MALDI-TOF) mass spectrometry (Sequenom Inc., San Diego, CA, USA). Assays for all SNPs were designed using Spectro DESIGNER software (Sequenom Inc., San Diego, CA, USA) and genotyped using the iPLEX assays (www.sequenom.com/iplex accessed on 3 November 2022) as described previously [26].

The innate panel SNPs were typed using tetra-primer Amplification refractory mutation system (ARMS) PCR following the method of Ye et al. [27] with modifications optimized for our SNP panel.

Briefly, primers for each selected polymorphism were designed using primer design software available at http://cedar.genetics.soton.ac.uk/public_html/primer.html accessed on 3 November 2022. The software has been optimized to include two deliberate mismatches in the inner primer sets at 3′ termini and −2 bases from 3′ terminal to aid in the allele specificity. Each PCR reaction was carried out in a total volume of 10 μL containing 30 ng of template DNA, 10 pmol of each outer and inner primers, 200 mM of dNTPs, appropriate. concentration of Mgcl_2_ (2.5 −3.5 nm), 20 mM Tris-Cl pH 8.4, 50 mM KCl, 0.05% (*v*/*v*) W1 and 0.5 Units of thermostable Taq polymerase. The PCR conditions included 2 min at 95 °C, 1 min annealing (annealing temperatures different according to primers) and 1 min extension (72 °C), and additional two minutes extension at 72 °C at the end of 35 cycles. Representative images of all genotyped polymorphisms are available in Figure S1A–O.

To confirm that ARMS PCR detection of genotypes matches up with Sequenom genotyping, we selected a SNP (rs3212220) form *IL12.* This SNP was which was been genotyped on the Sequenom platform as well as ARMS-PCR was carried out for all the individuals of the control group and full concordance was observed (Table S1). *LTA4H* gene SNP rs17525495 was typed using allelic discrimination assay (catalog number: 4351379, Applied Biosystems) using manufacturer’s instructions.

### 2.4. ELISA for Serum Cytokine Measurement

Serum collected from the abovementioned cohort was quantified for the level of circulating cytokines by using Enzyme-linked Immunosorbent assay (ELISA) using cytokine kits following the manufacturer’s instructions. The unknown values were extrapolated from a standard curve within the linear range.

### 2.5. Statistical Analysis

Hardy-Weinberg equilibrium was assessed in cases and controls for all tested variants to ensure that the samples were within allelic population equilibrium by using Haploview v 4.2 (http://www.broad.mit.edu/mpg/haploview/ accessed on 3 November 2022). A stringent cut off offered by the Haploview v 4.2 was used to perform further analysis which was used as a filtering criterion which included the following parameters: Minimum genotype = 75% and minimum minor allele frequency 0.0010) and HWE controls *p* > 0.05. The samples and SNPs failing this test were not selected for further analysis. PLINK v 1.07 (http://pngu.mgh.harvard.edu/purcell/plink/ accessed on 3 November 2022) was used to correct for multiple comparison, using Bonferroni methods, *p*-value after correction considered significant in the validation panel. Haplotype block generation was performed using the algorithm by Gabriel et al., 2002 [28] implemented in the Haploview software (http://www.broad.mit.edu/mpg/haploview/ accessed on 3 November 2022) which was also used for initial association testing. Genetic association testing was done using a 2 × 2 contingency table. Odds ratio, two tailed *p*-value was calculated for alleles using GraphPad Prism (version 5.00 for Windows, Graph Pad Software, San Diego, CA, USA, www.graphpad.com accessed on 3 November 2022). A two-tailed *p* value < 0.05 was considered statistically significant. The Odds ratio was confirmed by PLINK v 1.07, using a general model with fisher’s exact test options. Multidimensional scaling using pairwise identity-by-state distances which was inferred based on genotypes of the 34 SNPS of the cytokine was carried out in. PLINK v 9.0. Raw Distances were plotted on a 3D plot in R using the ‘rgl’ package.

For gene-gene interaction analysis we applied semi-exhaustive testing for pairwise interaction using PLINK v 1.07. The --fast epistasis along with --case-only option was used for this purpose. This has been hailed as a powerful approach by some workers. It provides a logistic regression test for interaction. This analysis exploits the fact that under certain conditions an interaction term in logistic regression equation corresponds to dependency or correlation between relative predictor variables within the population of cases. It uses an allelic model for both main effects and interactions and genotypes are not correlated.

### 2.6. SNP Targeted Pathway Analysis

We performed a SNP targeted pathway analysis using the PANOGA protocol [29] as shown by Bakir-Gungor et al. [30]. The PANOGA protocol uses the association information in terms of *p*-value and creates files that can be used as input files in Cytoscape [31] application of the JActiveModules [32], which takes the genes containing the SNPs information and extrapolates it to the human whole human protein-protein interaction network and derives network and sub-network based on the input genes in the query. The JActiveModules output consisting of networks is then used as an input in ClueGo app [33] in which one can look for gene annotations from various sources including the KEEG, WikiPathways [34], GO database for immunological, biological, molecular and other networks that has been used to visualize the pathways to the gene interaction scale. We chose the WikiPathways to visualize the genes in the results.

## 3. Results

### 3.1. The Study Population Was Devoid of Population Stratification

**Figure 1:**
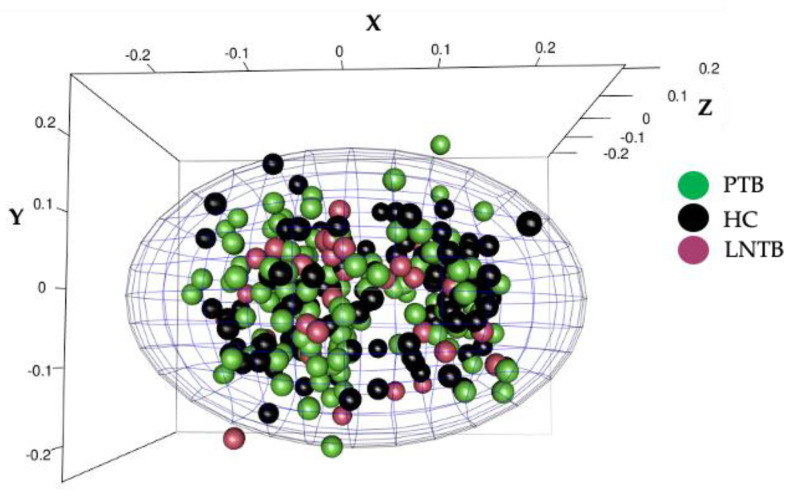
**The study groups show homogeneity forming a tight cluster showing no stratification in the samples.** Figure depicts a three-dimensional plot for checking population stratification among the study groups i.e., PTB (green spheres), LNTB (Purple Spheres) and HC (green Spheres). Raw Hamming distances as multidimensional scaling (MDS) co-ordinates are plotted on X, Y and Z-axes, to visualize genetic distance between the study groups.

False-positive associations can arise as a result of population stratification [35]. To investigate any hint of population-substructure, the self-reported ethnicity of each subject and his/her parents was carefully considered. To rule out population stratification a Multidimensional scaling (MDS) analysis was carried out on the genotyping data from the groups, which generated a compact cluster, without separating, indicating that population of patients (PTB and LNTB) and control subjects (HC) were homogenous with no sub-structures (Figure 1).

### 3.2. Cytokine Genetic Variants Show Significant Allelic and Haplotypic Association in PTB and Not in LNTB

For analysis of the genetic association of the cytokine variants, we initially constituted a discovery panel of 39 SNPs listed in Table S2. We genotyped these cytokine polymorphisms in 110 PTB cases, 78 HC and 35 LNTB cases. A Combined (PTB + LNTB) comparison (Table 1) was followed by a separate PTB and LNTB comparison for association (Table 1), with the aim to identify the SNPs- linked to differential susceptibility to PTB and LNTB. 26 SNPs for PTB, 23 SNPs for LNTB and 24 SNPs combined, passed the filtering criteria, and were analyzed for allelic association are listed in Table S3. The significant and borderline significant associations are enlisted in Table 1.

When considering PTB and LNTB cases together for analysis (Combined) we could identify only one variant from *IL10* gene rs1878672 of significance while certain others showed trend for association such as rs746868 of *TNFB*, rs1143643 *IL1B* and rs419598 of *IL1RA.* From the PTB only analysis we could identify only one variant from *IL10* gene rs1878672 of significance with C allele showing 3.4-fold risk of developing PTB. While certain others which showed trend for association in all TB group such as rs746868 of *TNFB* (1.6-fold risk), rs1143643 of *IL1B* (3.2-fold risk) showed association with PTB group, indicating that disease type has a bearing on the susceptibility to TB. From the LNTB only analysis we could identify only variant from *IL6* gene, rs1548216 of significance with C allele showing a 4.4-fold risk of developing LNTB.

Analysis of the gene structure in the combined analysis revealed two haplotype blocks formed by SNPs in *TNFB* and *IL18* (Table 2). The haplotypic frequency among case and controls did not differ significantly for both PTB and combined groups (Table 2). While the combined and PTB analysis showed two combinations each for *TNFB* and *IL8*, LNTB showed four combinations for *TNFB*, the haplotypic frequency of one of which, the TTC showing 4.4-fold risk of developing LNTB (Table 2). No multiple corrections were carried out at this stage in the analysis. The aim was not to prematurely discard SNPs and select them for further validation in a larger sample size.

After, discovering that cytokine gene polymorphism associations with PTB and to a lesser extent in LNTB, we wanted to independently validate, these findings before making a conclusion. We selected, 15 SNPs for the validation panel from eight cytokine genes (Table S2b). Seven of these genes and SNPs were selected association in the discovery panel (Table 1, Table S3) and the eight SNPs found from our previous study on 25 SNPs (Table S2b) from six cytokine genes [26], which achieved a replication sample size of 160 PTB cases, 50 LNTB cases and 265 controls, giving the validation panel a 91% power of study to detect an odds ratio of 1.8 and above for nine cytokine SNPs (Table S2b) [36].

Upon analysis of 15 cytokine SNPs after applying Bonferroni’s correction for multiple testing, (Table 3), for PTB we found association for *IFNG* at rs1861493, *IL4* at rs2853694 and *IL12* at rs3212220 after correction for multiple testing. This replicated our previous findings about cytokine gene polymorphisms increasing the risk for PTB [26]. In contrast, for LNTB two variants rs2070874 of *IL4* and rs2853694 of *IL12*, showed significance but these associations were lost after correction for multiple testing. (Table 3). Interestingly, out of the 7 SNPs from the discovery panel which was significantly associated in (Table 1 and Table 2), only rs3024498 of *IL10* gene achieved a borderline significance (*p* = 0.07) (Table S4a) and the rest was not replicated in the validation cohort for LNTB. This could be related to a lower replication sample size of the validation panel for LNTB. Therefore, these SNPs need larger sample size for validation. Here, we could validate our previous cytokine gene association findings [26]. This analysis showed that cytokine genetic variants increase the risk of PTB but not LNTB. These observations add value to the argument that genetic polymorphisms play a critical role in manifestation of TB as pulmonary or extra-pulmonary TB.

### 3.3. Gene-Gene Epistatic Interaction Analysis Reveals a Higher Risk for Cytokine Genes Majorly in PTB and Not LNTB

After determining that cytokine gene polymorphisms contributed to increased risk for PTB susceptibility, we applied semi-exhaustive epistatic testing for pairwise interaction among the significantly associated SNPs from a previous panel [26] and the current cytokine gene validation panel to understand their genetic interaction. Thirteen significant interactions were identified and are enlisted in Table 4. Interestingly, the *IL4* locus showed interaction in LNTB as well, highlighting a critical role for this SNP in the north Indian population (Table 4). This approach also identified some SNPs which were not associated in the single locus analysis. *IL1RA* emerged as the gene having a significant interaction with *IL12*, *IL4* and *TNFB* genetic variants. Most of the *IL1RA* interactions were protective with odds ratio <1. Only one interaction between its own SNP was showing an eight-fold risk (*p* = 3.066 × 10^−5^). This interaction could be important in defining the genetic susceptibility to TB. The other important player was *IL4* which showed interaction with variants of *IL12*, *IL1RA* and *TNFB*. Interestingly, all the interactions of IL4, an anti-inflammatory cytokine with other proinflammatory cytokines such as *IL12* and *TNFB* showed a very high risk (18-fold risk) with very highly significant *p*-values. These genetic interactions enabled us to test the hypothesis that the disease outcome in tuberculosis can be due to interaction of the cytokine gene polymorphism. Also, many of the loci identified here were not significant in single variant association analysis. This analysis confirmed that cytokine gene polymorphisms affect the outcome of PTB more than LNTB, adding evidence to support the role of genetic polymorphisms in differential disease manifestation in TB.

### 3.4. Lack of Major Association of Cytokine Levels with Genotypes in LNTB

**Figure 2:**
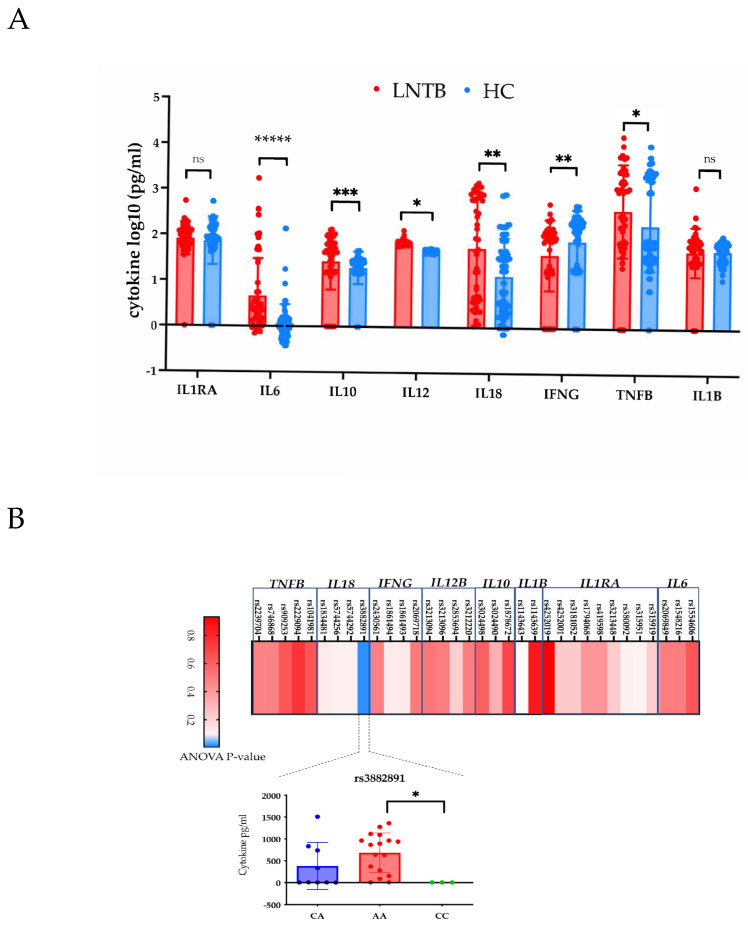
**Cytokine levels have limited correlation with genotypes in LNTB.** (**A**) Increased cytokine levels in LNTB (n = 50, red) vs HC (n = 84, Blue). The groups were compared using a multiple Mann-Whitney test. ***** *p* < 0.00001, *** *p* < 0.001, ** *p* < 0.01, * *p* < 0.05, (**B**) Heatmap of overall ANOVA *p*-Values of 34 SNPs in the cytokine genes with the corresponding cytokine levels. Shades of Red depict non-significant *p*-values and shades of blue significant *p*-value. Only significant variant, *IL18* at rs3882891 is shown with the genotypes with their respective levels, where AA genotype individuals are highest IL18 producers and compared to CA and CC (*p* < 0.05) genotypes.

We have previously shown that cytokine levels are affected by their genotypes, and individuals with a certain genotype secrete more of less of cytokines in their serum in people with PTB [37]. Since, we observed such stark differences in the association of cytokine gene polymorphism in PTB and LNTB, we carried out a similar analysis for the LNTB samples in this study. Overall, LNTB showed higher levels of the cytokine as compared to the healthy controls (Figure 2A). Out of 34 SNPs tested, none of the cytokine genotypes except for *IL8* at rs3882891 showed any significant difference in cytokine levels as governed by their genotype (Figure 2B), lending credibility to a major role of cytokine gene polymorphism in PTB but not LNTB.

### 3.5. Innate Immunity Related Genes Are Majorly Associated with LNTB and Not PTB

Innate immunity forms the first line of defense and multiple of innate immune genes have been implicated in susceptibility to PTB in various populations of the world where TB is endemic [22]. Since we observed such stark differences in cytokine gene polymorphisms, we hypothesized that a number of these gene polymorphisms would be PTB or LNTB specific. Widely studied polymorphisms were selected on for the study, as it would offer us a great comparative insight with other world populations for TB susceptibility. The allelic association of the innate genes is listed in Table 5.

*P_2_RX_7_* gene showed a 7-fold risk for: −762 T/C (rs2393799) C allele for the development of LNTB this association was marginally associated with risk of developing PTB. For rs37511431 we didn’t detect any association. Out of three studied variants of the *VDR* gene variants i.e., *Fok*I (rs2228570), *Taq*I (rs731236), *Bsm*I (rs1544410), rs1544410 was found to be not polymorphic (presence of only one allele detected), rest of the two polymorphisms were not found to be associated with TB (both PTB and LNTB) risk or protection in this population. We did not find any association between either PTB or LNTB and *IRGM* genetic variant rs9637876 (Table 5). The results indicated that *NRAMP1/SLC11A1* gene polymorphic variants may not be associated with the susceptibility to TB in the studied population. In fact, we could detect the presence of only a single genotype in all cases and controls; a CC genotype for rs3731865 and a heterozygous AG genotype for rs17235409. No haplotypes were observed. G allele of the *TLR2* genetic variant rs6265786 (Arg677Trp) of showed a high risk for PTB but not for LNTB. Similar results obtained for rs4986790 of *TLR4* gene where the G allele of shows a 2-fold risk of development of PTB. For *DCSIGN* (*CD209*) in PTB cases, with allele ‘A’ of rs4804803 was overrepresented in cases as compared to healthy controls showed a very significant association posing a 4-fold risk for developing PTB in north Indians and a 1.9-fold risk in LNTB cases (Table 5). None of the two tested *NOS2A* variants i.e., rs2274894 and rs7215373 showed any association either in PTB or LNTB, although the variant rs7215373 showed marginal association for both PTB and LNTB. Of the three studied three polymorphisms in the *LTA4H* gene rs1978331, rs2660898, and rs17525495, none of the variant showed association in either PTB or LNTB. *LTA4H* gene polymorphisms have been shown to provide heterozygous protection, implying that a heterozygous genotype is protective from TB [12]. When we compared the heterozygous genotypes vs the homozygotes as proposed by Tobin et al., we observed, that out of three typed variants, rs1978331 have a protective association (odds ratio < 1) in combined and LNTB but not in PTB (Table 6). Similar, odds were observed between the haplotype of rs1978331-rs2660898, where when both the SNPs are heterozygous, they are borderline protective for LNTB and not PTB (Table S4). We have previously shown that, *SP110* gene polymorphisms were associated with risk of LNTB and not PTB in this population [19]. To continue exploring this gene in an independent cohort, we genotyped, *SP110* variants rs6436915, rs1346311, rs7580900. As shown previously and none of these showed any allelic associations in PTB (Table 5). Due to limited independent samples for LNTB, these were not genotyped.

Since, we observed a uniform *TLR* gene polymorphism risk for both PTB and LNTB, we also genotyped four *TLR8* gene polymorphisms, as their genetic association has been shown to be important for outcome of TB. Uniquely, *TLR8* is located on the X chromosome, so the males as they have only one copy of the X chromosome, would be hemizygous. Analyzing the risk in males and females separately, revealed a higher risk for males as expected carrying A allele for rs3788935 (17-fold risk), rs3761624 (4-fold risk). A risk for female population was also detected for rs3761624 which was lost after multiple corrections testing. If A allele is risk factor for males as they carry only one copy a corresponding homozygous phenotype can be a risk factor for females too as depicted by rs3761624. The sample size of LNTB group (*n* = 50) was limited for a stratified analysis by sex, so it was not carried out for LNTB (Table 7). Interestingly, in a case vs control analysis not stratified by sex *TLR8* PTB showed an increased risk for 3 (rs3788935, rs3761624, rs3764880) and LNTB 2 (rs3761624, rs3764879) among the four variants typed (Table S5).

### 3.6. Pathway Analysis Reveals an Apoptotic Axis for LNTB and a Cytokine Axis for PTB

**Figure 3:**
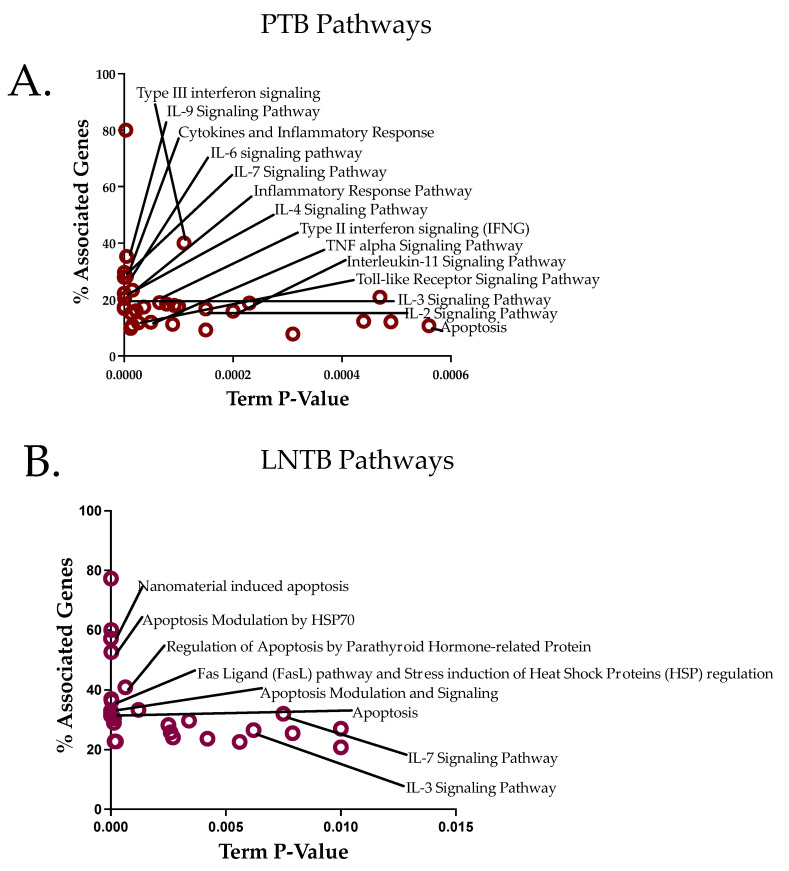
PTB and LNTB risk associated SNP related genes enrich in different pathways. Pathways enriched in PTB (**A**) and LNTB (**B**) through a pathway analysis based on the SNPs with significant *p*-values. The modules obtained from JActivemodules were used for enrichment in ClueGO App in Cytoscape. Two metrics, %Associated genes (proportion of genes in the pathway from the network of all genes) and Term *p*-value (enrichment *p*-value) is plotted here to show the abundance of the pathway with all the input genes and the *p*-value for the GO term obtained from WikiPathways.

SNP association and their respective *p*-values from the study was used as an input to identify associated modules from a protein-protein interaction network, which was used to identify the associated pathways and the results obtained were subjected to a gene-ontology annotation using WikiPathways [29,30]. All the SNPs that are significantly associated in this study and previous studies on this population [19,26] were considered. An abundance for cytokine pathways was seen for PTB (Figure 3A), while abundance of apoptosis modules was seen for LNTB (Figure 3B). This pathway level difference is in line with the genetic association findings presented above, showing these differential genetic association could contribute to differential pathway activation and hence activate the immune response distinctly. This adds another level of evidence of differential host genotype being responsible for different manifestation of PTB and LNTB.

## 4. Discussion

Human genetic diversity is hugely impacted by co-evolving pathogens such as *Mtb* [38]. Candidate gene studies using the case—control design provides one of the most direct means of identifying human genetic variants that currently impact on susceptibility to infectious disease. Such information would help improve the understanding of disease pathogenesis and disease resistance at an individual level, that could inform targeted intervention strategies based on their genotype as has been successfully implemented [12].

Several studies have shown that TB susceptibility has a genetic component (summarized in [22,39], but comparative studies on genetic susceptibility to different forms of TB are limited [21,40]. Such studies can provide insight into the role genetic polymorphisms in different manifestations of TB. Even rarer are studies on genetic susceptibility to EPTB. Some of the studied forms of EPTB have been involving multiple sites [16], LNTB [19,41,42], TB meningitis [42,43], intestinal TB [44], bone [42] and pleural [42].

In this study, we aimed to do a comparative study between PTB and LNTB to investigate differential genetic associations between PTB and LNTB. We tested several candidate gene polymorphisms, never investigated before, as associated with differential TB susceptibility (Table S1). In addition, we validated susceptibility loci previously identified in other populations [22,39] and our previous studies [19,26]. This is important as ethnic validation of commonly reported genetic variants in different populations is desirable. In total, 63 polymorphisms across 23 genes were selected and genotyped from both the innate and adaptive immune branches of immunity to TB in the north Indian population and their allele frequencies compared and linkage disequilibrium (LD) and haplotypes investigated. Thus, we have employed a comprehensive coverage of SNPs and genes to compare the genetic susceptibility differences between PTB and LNTB.

In our study, genetic variants in the cytokine were validated to be significant risk factors PTB (Table 3). We also showed that a significant gene—gene interaction among cytokine SNPs may further accentuate the importance of the identified SNPs in governing the genetic susceptibility to PTB. Interestingly, we didn’t find significant cytokine gene polymorphisms associated with LNTB. The important difference was lack of a major association between cytokine SNPs and serum cytokine levels in LNTB, which has been shown to be associated with PTB in multiple studies [37,45,46,47]. The difference between such association clearly shows that there are distinct genetic coordinates for with PTB and LNTB susceptibility. Highly enriched cytokine pathways in PTB and limited in LNTB (Figure 3) add strength to this argument.

Interestingly the innate immunity genes, *P2RX7* [48] and *DCSIGN* [49,50,51], which are critical for immune response to *Mtb*, were risk factors for both PTB and LNTB, as expected. *P2RX7* has been very widely studied as risk factor for both PTB and EPTB. Macrophages from patients with loss of function homozygous allele for rs3751143, could not kill *Mtb* in vitro in EPTB [16]. We didn’t observe any association with this variant in our study. Interestingly, another functional variant rs2393799, showed an increased risk for both PTB and LNTB, but the risk was much higher for LNTB (7-fold as compared to 1.5-fold for PTB). *P2RX7* is known to have a role in apoptosis of *Mtb* infected macrophage [24]. Similar theme was seen for *SP110* gene for which we have previously identified a risk for rs1427294 of in LNTB but not pulmonary TB [19]. Recently, this gene has been shown to inhibit apoptosis of infected macrophages, thereby resisting *Mtb* infection [23]. This in conjunction of identifying more apoptotic pathways do suggest that the apoptotic axis may be important in LNTB. The other genetic variants of importance in the *LTA4H* gene showed heterozygous protection in LNTB and not PTB (Table 6). So, we show in the current study that genetic variations in the innate immune genes have a closer relation to development of LNTB, whereas the cytokine genetic variants have little influence and associations in LNTB. Similarly, among the pattern recognition receptors, *TLR2* and *TLR4* showed risks for PTB but not for LNTB. *TLR8* genetic variants showed risk for both PTB and LNTB with more risk for males in PTB. This adds to the theme of differential association between PTB and LNTB, *TLR*s variants have been shown to be critical risk factors for TB [52,53,54,55] but have not been studied for LNTB. Although, limited by sample size there appears to be differences in TLR gene polymorphisms in PTB and LNTB.

Similar to our study a few other studies have shown a selective genetic association with EPTB, for example, like our study (Table 6) Yang et al. show that *LTA4H* gene polymorphism rs1978331 and rs2540474, are only risk factor with EPTB and not PTB in Han Chinese population [42]. Similarly, a GWAS could identify 4 loci that were only associated with EPTB and not PTB [21]. These studies support the differential nature of genetic polymorphism in EPTB, which is distinct from PTB. Similar studies are warranted for validation in a larger sample size and in multiple populations to test whether genetic polymorphism can associate with various forms of tuberculosis. The limitation of the study is that for certain polymorphisms we could not achieve a good sample size and thus the results need to be validated in a larger sample size.

## 5. Conclusions

Our study contributes to the growing knowledge that PTB and EPTB manifestations have a genetic basis. The highlight of our study was finding more polymorphic cytokine genes in PTB and more polymorphic apoptosis/innate genes in LNTB.

## Figures and Tables

**Table 1:** Comparative allelic association statistics of cytokine variants in the discovery group between PTB and LNTB.

Gene	SNP (rsID)	Risk Allele	Case, Control Frequencies			Odds Ratio (CI)			*p*-Value		
			PTB (n = 110)	LNTB (n = 35)	Combined (n = 145)	PTB	LNTB	Combined	PTB	LNTB	Combined
*TNFB*	rs746868	C/C/C	0.650, 0.543	0.589, 0.543	0.637, 0.543	**1.6 (1.0–2.4)**	NA	**NA**	**0.0442**	0.5545	**0.0647**
*IL10*	rs1878672	C/G/C	0.937, 0.812	0.250, 0.188	0.894, 0.812	**3.4 (1.6–7.2)**	NA	**2.0 (1.1–3.6)**	**0.0007**	0.3487	**0.0271**
*IL1B*	rs1143643	G/A/G	0.935, 0.817	0.286, 0.183	0.887, 0.817	**3.2 (1.6–6.6)**	NA	NA	**0.001**	0.1174	**0.0635**
*IL1RA*	rs419598	C/T/C	0.418, 0.264	0.796, 0.736	0.369, 0.264	**2 (1.2–3.4)**	NA	NA	**0.0079**	0.401	**0.054**
*IL6*	rs1548216	G/C/C	0.985, 0.978	0.089, 0.022	0.031, 0.022	NA	**4.4 (1.0–19)**	**NA**	0.6447	**0.032**	0.5846

Significant *p*-Values and borderline *p*-Values are shown in bold.Significant odds ratio is indicated in bold. rsID: dbSNP, the SNP database (http://www.ncbi.nlm.nih.gov/projects/SNP accessed on 3 November 2022); Abbreviations: CI: Confidence Interval, NA: The odds ratio were not calculated as the *p*-Value was not significant.

**Table 2:** Comparative Haplotypic association statistics of cytokine variants in the discovery group between PTB and LNTB.

Gene	Block	Haplotype			Case, Control Frequencies			*p*-Value		
		PTB	LNTB	Combined	PTB	LNTB	Combined	PTB	LNTB	Combined
***IL18***	Block 1									
	TG	0.907	0.884	0.905	0.926, 0.880	0.893, 0.880	0.919, 0.880	0.153	0.8036	0.211
	CC	0.093	0.116	0.095	0.074, 0.120	0.107, 0.120	0.081, 0.120	0.153	0.8036	0.211
***TNFB***	Block 2									
	GC/GCC	0.609	0.281	0.601	0.650, 0.549	0.313, 0.268	0.629, 0.548	0.0568	0.5305	0.1146
	TG/GTC	0.383	0.269	0.387	0.343, 0.441	0.241, 0.280	0.358, 0.442	0.0637	0.5736	0.098
	TTC		0.011			0.036, 0.001			**0.0303**	
	TTG		0.433			0.411, 0.442			0.6911	

Multiallelic haplotype blocks are depicted. Block 1 consisted of rs5744256, rs1834481 for *IL18* gene; For *TNFB* gene in Block 2 the variants consist of rs2239704, rs746868 for PTB and Combined group and rs2239704, rs909253 and rs746868 for LNTB. The rsIDs listed are in the same order as they appear in the haplotype blocks. Biallelic combination of haplotype showed two combinations for PTB and Combined and four combinations for LNTB. Significant *p*-Values are indicated in bold. ^a^ Odds Ratio (95% CI) = 4.4 (1.0–19).

**Table 3:** Comparative allelic association of the cytokine gene variants in the validation panel between PTB and LNTB.

Gene	SNP (rsID)	Risk Allele	Case, Control Frequencies			Odds Ratio (CI)			*p*-Value			P-Bonferroni		
			PTB	LNTB	Combined	PTB	LNTB	Combined	PTB	LNTB	Combined	PTB	LNTB	Combined
***IFNG***	rs1861493	A	0.935, 0.858	0.148, 0.142	0.912, 0.858	**2.4** **(1.3** **–4.2)**	1.1 (0.62–1.8)	**1.7** **(1.1–2.7)**	**0.002**	0.853	**0.0197**	**0.013**	1	0.138
***IL1RA***	rs4252019	C	0.974, 0.934	0.090, 0.066	0.962, 0.934	**2.6** **(1.1–6.0)**	1.4 (0.72–2.7)	1.8 (0.93–3.4)	**0.018**	0.314	0.078	0.159	1	0.71
***IL4***	rs2070874	T	0.306, 0.239	0.847, 0.761	0.280, 0.239	**1.4** **(1.0–2.0)**	**1.7** **(1.1–2.9)**	1.2 (0.90–1.7)	**0.048**	**0.027**	0.194	0.303	0.2436	1
***IL12***	rs2853694	A	0.624, 0.508	0.620, 0.508	0.608, 0.508	**1.6** **(1.2–2.2)**	**1.6** **(1.1–2.3)**	**1.5** **(1.1–2.0)**	**0.002**	**0.018**	**0.004**	**0.017**	0.1624	**0.032**
	rs3212220	T	0.409, 0.297	0.355, 0.297	0.394, 0.297	**1.6** **(1.2–2.2)**	1.3 (0.8–1.9)	**1.5** **(1.2–2.1)**	**0.002**	0.186	**0.003**	**0.017**	1	**0.031**

Abbreviations: SNP: single nucleotide polymorphism; rsID: dbSNP, the SNP database (http://www.ncbi.nlm.nih.gov/projects/SNP accessed on 3 November 2022); Two tailed *p* < 0.05 was considered significant. Significant *p*-Values and odds ratios are indicated in bold CI: Confidence Interval; *p*-value is derived from a chi-square test; P-Bonferroni: *p*-value after applying Bonferroni correction for multiple testing. For the validation the sample size PTB (*n* = 160), LNTB (*n* = 50), HC (*n* = 265).

**Table 4:** Comparison of Epistatic interactions in cytokine genes between PTB and LNTB.

CHR1	Gene	SNP1	CHR2	Gene	SNP2	OR_INT	STAT	*p*-Value	TB Form
2	***IL1RA***	rs1794068	2	***IL1RA***	rs3213448	0.07834	21.47	3.60 × 10^−6^	PTB
2	***IL1RA***	rs1794068	2	***IL1RA***	rs3181052	0.08759	22.68	1.92 × 10^−6^	PTB
2	***IL1RA***	rs1794068	5	***IL12***	rs3213119	0.04328	24.7	6.69 × 10^−7^	PTB
2	***IL1RA***	rs1794068	5	***IL12***	rs3213096	0.06435	25.68	4.03 × 10^−7^	PTB
2	***IL1RA***	rs1794068	6	***TNFB***	rs3093542	0.05699	26.41	2.76 × 10^−7^	PTB
2	***IL1RA***	rs315951	2	***IL1RA***	rs9005	8.265	17.38	3.07 × 10^−5^	PTB
2	***IL1RA***	rs315951	5	***IL4***	rs2243266	0.1028	17.66	2.64 × 10^−5^	PTB
2	***IL1RA***	rs315951	5	***IL12***	rs3213119	0.008753	31.27	2.25 × 10^−8^	PTB
2	***IL1RA***	rs315951	5	***IL12***	rs3213096	0.007691	32.86	9.92 × 10^−9^	PTB
2	***IL1RA***	rs315951	6	***TNFB***	rs3093542	0.008263	32.32	1.31 × 10^−8^	PTB
5	***IL4***	rs2070874	5	***IL12***	rs3213119	18.18	29.85	4.66 × 10^−8^	PTB
5	***IL4***	rs2070874	5	***IL12***	rs3213096	18.33	30.69	3.03 × 10^−8^	PTB
5	***IL4***	rs2070874	6	***TNFB***	rs3093542	18.86	31.75	1.76 × 10^−8^	PTB
5	IL4 *	rs2070874	5	***IL12***	rs3213096	16.71	37.64	8.52 × 10^−10^	PTB
5	IL4 *	rs2070874	5	***IL12***	rs730690	5.056	16.02	6.26 × 10^−5^	PTB
5	IL4 *	rs2070874	5	***IL4***	rs2243266	5.007	23.61	1.18 × 10^−6^	LNTB

Abbreviations: CHR1: Chromosome of 1st SNP, SNP1: Identifier of 1st SNP; CHR2: Chromosome of 2nd SNP, SNP2: Identifier of 2nd SNP; OR_INT: Odds ratio for interaction; STAT: Chi-square,1-df (degree of freedom); *p*-Value: Asymptomatic *p*-value, * the interaction for the validation panel.

**Table 5:** Comparative Allelic association between various innate gene polymorphisms between PTB and LNTB.

Gene	db SNP rsID/Other Name	Gene Location	Risk Allele	Case, Control Frequencies		Chi Square		*p*-Value		Odds Ratio	
										(CI)	
				PTB	LNTB	PTB	LNTB	PTB	LNTB	PTB	LNTB
***P2RX7***	rs2393799/−762T/C	Exon	C	0.707, 0.611	0.917, 0.611	3.892	19.23	**0.048**	**1.15 × 10^−5^**	**1.5 (1–2.4)**	**7 (2.6–18.4)**
	rs3751143/1513 A/C	Exon	C	0.574, 0.525	0.574, 0.525	0.816	0.079	0.3664	0.7791	1.2 (0.79–1.7)	1.1 (0.5–2.2)
***VDR***	rs2228570/*FokI*	Exon	G	0.505, 0.472	0.552, 0.472	0.436	1.151	0.5	0.288	ND	ND
	rs731236/*TaqI*	Exon	C	0.893, 0.861	0.895, 0.525	0.849	0.298	0.357	0.5849	ND	ND
	rs1544410/*BsmI*	Exon	Only GG Genotype observed								
***IRGM***	rs9637876/−261T/C	Alu sequence	C	0.306, 0.255	0.272, 0.255	1.53	1.2	0.2161	0.515	ND	ND
***TLR2***	rs6265786/Arg 299 Trp	intron	G	1.000, 0.958	0.979, 0.958	9.53	0.471	**0.002**	0.4925	**20.92 (1.2–369.3)**	2.1 (0.25–17.25)
***TLR4***	rs498670/Asp 299 Gly	Intron	G	0.223, 0.125	0.05, 0.125	6.09	2.622	**0.0135**	0.1054	**2.1 (1.2–2.5)**	0.11 (0.10–1.3)
***NOS2A***	rs7215373	Intron	T	0.481, 0.405	0.514, 0.400	2.732	2.914	0.0983	0.0878	1.3 (0.94–1.95)	1.6 (0.94–2.65)
	rs2274894	3’UTR	T/G	0.213, 0.171	0.05, 0.125	1.348	0.45	0.2456	0.5022	1.3 (0.83–2.06)	1.3 (0.63–2.6)
***SLC11A1/NRAMP1***	rs3731865	Exon	Only CC genotype observed								
	17235409	Intron	Only AG genotype observed								
***SP110***	rs6436915	Intron	T	0.421, 0.347	0.417, 0.350	2.766	1.038	0.0963	0.3082	ND	ND
	rs1346311	Intron	T	0.105, 0.104	0.959, 0.900	0.001	2.516	0.9701	0.1127	ND	ND
	rs7580900	Intron	T	0.531, 0.473	0.554, 0.472	1.613	1.471	0.204	0.2252	ND	ND
***DCSIGN (CD209)***	rs4804803/−336 A/G	Promoter	A	0.745, 0.380	0.534, 0.380	55.47	4.52	**9.48 × 10^−14^**	**3.34 × 10^−2^**	4.0 (2.6–6.0)	1.9 (1.0–3.4)

SNP, single nucleotide polymorphism; Abbreviations: dbSNP, the SNP database (http://www.ncbi.nlm.nih.gov/projects/SNP accessed on 3 November 2022); Two tailed *p* < 0.05 was considered significant. *p* value from a chi-square test, significant *p*-Value and Odds Ratios are indicated in bold, CI: Confidence Interval.

**Table 6:** Allelic association for the *LTA4H* gene with and without the heterozygosity model.

SNP	Groups	N	Genotype Frequencies			Allele Frequencies		Without Heterozygosity Model		Heterozygosity Model		
										(01 vs. 00 + 11)		
			00	01	11	0	1	OR (CI )	*p*-Value	OR (CI)	*p*-Value	Padj
rs1978331	All TB	185	0.26	0.44	0.30	0.48	0.52	1.02 (0.73–1.41)	0.9209	0.55 (0.34–0.87)	**0.013**	**0.039**
T = 0, C = 1	PTB	135	0.25	0.46	0.29	0.48	0.52	1 (0.70–1.43)	0.9738	0.60 (0.36–0.99)	0.076	0.168
	LNTB	50	0.3	0.38	0.32	0.49	0.51	1.04 (0.65–1.66)	0.8533	0.43 (0.22–0.85)	**0.018**	**0.037**
	Control	120	0.18	0.59	0.23	0.47	0.52					
rs2660898			TT	TG	GG							
T = 0, G = 1	All TB	185	0.17	0.41	0.42	0.38	0.63	1.21 (0.85–1.7)	0.2761	0.76(0.48–1.2)	0.283	0.849
	PTB	135	0.15	0.42	0.44	0.36	0.64	1.1 (0.76–1.61)	0.5822	0.77(0.46–1.3)	0.309	0.927
	LNTB	50	0.22	0.4	0.36	0.43	0.57	1.5(0.93–2.44)	0.093	0.75(0.38–1.5)	0.496	1.488
	Control	120	0.09	0.48	0.43	0.33	0.67					
			TT	TC	CC							
rs17525495	All TB	185	0.06	0.24	0.69	0.19	0.82	1.33(0.83–2.1)	0.238	0.95(0.53–1.7)	0.8827	2.6481
T = 0, C = 1	PTB	135	0.05	0.25	0.65	0.19	0.81	1.31(0.78–2.2)	0.298	1.1(0.60–2.1)	0.753	2.259
	LNTB	50	0.08	0.18	0.62	0.19	0.80	1.38(0.72–2.65)	0.329	0.82(0.35–1.9)	0.831	2.493
	Control	120	0.03	0.24	0.73	0.15	0.85					

Abbreviations: OR: Odds ratio for the minor allele are shown. CI: Confidence Interval; Padj: *p*-Values adjusted by Bonferroni correction for multiple tests. *p*-Value from a chi-square test. For each SNP, OR was calculated for heterozygosity (01) versus homozygosity (00 + 11) for cases versus controls. Significant *p*-Values are indicated in bold.

**Table 7:** Sex-specific associations for *TLR8* gene variants in PTB.

db SNP rsID	Risk Allele	Males (Case = 75; Control = 70)				Female (Case = 50; Control = 54)			
		Case, Control Frequencies	*p*-Value	P Bonferroni	Odds Ratio (CI)	Case, Control Frequencies	*p*-Value	P Bonferroni	Odds Ratio (CI)
rs3788935	A	0.1102, 0.01	**0.0072**	**0.0288**	**17 (0.94–300)**	0.160, 0.106	0.1673	0.6692	1.6 (0.82–3.2)
rs3761624	A	0.2, 0.05	**0.012**	**0.048**	**4.3 (1.2–16)**	0.356, 0.244	**0.0423**	0.1692	**1.7 (1.0–2.9)**
rs3764880	A	0.107, 0.029	0.1012	0.4048	3.9 (0.81–19)	0.229, 0.149	0.0557	0.2228	1.7 (0.98–2.9)
rs3764879	C	0.558, 0.482	0.5934	2.3736	1.3 (0.61–2.6)	0.489, 0.440	0.3981	1.5924	1.2 (0.77–1.9)

## Data Availability

Data for the study has been summarized in multiple tables for the study. If needed, the data can be obtained from the first and corresponding authors upon reasonable request.

## Supplementary Materials

The following supporting information can be downloaded at: https://zenodo.org/record/7514176#.Y7sKTOzMLz0, Figure S1A–O: Representative ARMS-PCR gel images for the typed variants. Table S1: Concordance ARMS-PCR and Sequenom Genotyping for *IL12* at rs3212220. Table S2a: List of 39 Cytokine SNPS for the discovery Panel. Table S2b: List of 15 Cytokine SNPS for the Validation Panel. Table S3: Allelic association for the Discovery Panel. Table S4a: Validation results of the Discovery Panel. Table S4b: Haplotypic association following the heterozygosity model for *LTA4H* polymorphisms. Table S5: Comparative allelic association among PTB and LNTB for *TLR8* gene.
